# The impact of gastroesophageal reflux disease and its treatment on interstitial lung disease outcomes

**DOI:** 10.1186/s13075-024-03355-0

**Published:** 2024-06-25

**Authors:** A. Quinlivan, D. Neuen, D. Hansen, W. Stevens, L. Ross, N. Ferdowsi, S. M. Proudman, J. G. Walker, J. Sahhar, G-S. Ngian, D. Apostolopoulos, L. V. Host, G. Major, C. Basnayake, K. Morrisroe, M. Nikpour

**Affiliations:** 1 Department of Rheumatology, St Vincent’s Hospital (Melbourne), 35 Victoria Parade, Fitzroy, Victoria 3065 Australia; 2 Department of Medicine, The University of Melbourne at St Vincent’s Hospital (Melbourne), 41 Victoria Parade, Fitzroy, Victoria 3065 Australia; 3 Department of Rheumatology, Liverpool Hospital, Corner of Elizabeth St and Goulburn St, Liverpool, 2170 NSW Australia; 4 Rheumatology Unit, Royal Adelaide Hospital (Adelaide), Port Rd, Adelaide, South Australia 5000 Australia; 5 Discipline of Medicine, University of Adelaide (Adelaide), North Terrace, Adelaide, South Australia 5000 Australia; 6 Rheumatology Unit, Flinders Medical Centre (Adelaide), Flinders Drive, Bedford Park, South Australia 5042 Australia; 7 Immunology, Allergy and Arthritis Department, Flinders University (Adelaide), Sturt Road, Bedford Park, South Australia 5042 Australia; 8 Department of Rheumatology, Monash Health (Melbourne), 246 Clayton Rd, ClaytonVictoria, 3168 Australia; 9 Department of Medicine, Monash University (Melbourne), Wellington Rd, ClaytonVictoria, 3168 Australia; 10 School of Clinical Sciences, Monash University Faculty of Medicine Nursing and Health Sciences, Clayton, VIC Australia; 11 Department of Rheumatology, Fiona Stanley Hospital (Perth), 11 Robin Warren Drive, Murdoch, WA 6150 Australia; 12 Department of Rheumatology, Royal Newcastle Centre, John Hunter Hospital, 2 Lookout Rd, New Lambton Heights, New South Wales 2305 Australia; 13 Faculty of Medicine, University of Newcastle, University Drive, Callaghan, New South Wales 2308 Australia; 14 Department of Gastroenterology, St Vincent’s Hospital (Melbourne), 35 Victoria Parade, Fitzroy, Victoria 3065 Australia; 15 School of Public Health, University of Sydney, Edward Ford Building, Fisher Road, Camperdown, NSW 2006 Australia; 16 Department of Rheumatology, Royal Prince Alfred Hospital, 50 Missenden Road, Camperdown, NSW 2050 Australia

**Keywords:** Systemic sclerosis, Interstitial lung disease, Gastro-oesophageal reflux disease

## Abstract

**Background:**

To determine the relationship between gastroesophageal reflux disease (GORD) and its treatment and interstitial lung disease in patients with systemic sclerosis (SSc).

**Methods:**

SSc patients from the Australian Scleroderma Cohort Study (ASCS) were included. GORD was defined as self-reported GORD symptoms, therapy with a proton pump inhibitor (PPI) or histamine 2 receptor antagonist (H2RA) and/or the presence of reflux oesophagitis diagnosed endoscopically. The impact of GORD and its treatment on ILD features (including severity and time to ILD development) and survival was evaluated.

**Results:**

GORD was a common manifestation affecting 1539/1632 (94%) of SSc patients. GORD affected 450/469 (96%) of those with SSc-ILD cohort. In SSc-ILD, there was no relationship between the presence of GORD or its treatment and time to ILD development or ILD severity. However, GORD treatment was associated with improved survival in those with ILD (*p* = 0.002). Combination therapy with both a PPI and a H2RA was associated with a greater survival benefit than single agent therapy with PPI alone (HR 0.3 vs 0.5 *p* < 0.050 respectively).

**Conclusion:**

GORD is a common SSc disease manifestation. While the presence or treatment of GORD does not influence the development or severity of ILD, aggressive GORD treatment, in particular with a combination of PPI and H2RA, is associated with improved survival in those with SSc-ILD.

**Supplementary Information:**

The online version contains supplementary material available at 10.1186/s13075-024-03355-0.

## Background

Systemic sclerosis (SSc) is an autoimmune disease of the connective tissues characterised by pathologic mechanisms of vasculopathy, fibrosis and autoantibody formation [1]. The gastrointestinal system is the most commonly involved internal organ system in SSc with involvement of any area from mouth to anus [2]. Gastrointestinal (GI) disease has been reported in over 90% of patients, with gastroesophageal reflux disease (GORD) being the most frequently described symptom [2, 3]. Some studies suggest GORD may play a role in the development of interstitial lung disease (ILD) through the process of recurrent micro-aspiration [4, 5]. The exact pathogenesis of this association is unknown; however, it has been shown that exposure of the lung epithelium to gastric contents promotes airway epithelial cell fibrosis and enhanced fibroblast proliferation. Furthermore, in animal models, chronic aspiration resulted in parenchymal fibrosis [6, 7]. In SSc, the risk of aspiration of gastric contents is high due to the combination of oesophageal dysmotility, hypotensive lower oesophageal sphincter (LES) and delayed gastric motility [5, 8]. As such, it is not surprising that previous studies have demonstrated a relationship between the severity of GORD measured on pH-metry and manometry and the presence and severity of ILD measured by high resolution computed tomography (HRCT) of the chest and spirometry [5].

ILD is the leading cause of death in SSc [9] with some studies showing an association between the presence of GORD and /or oesophageal dysmotility and ILD [5, 10–12] Treatment of GORD with proton pump inhibitors (PPI) or histamine 2 receptor antagonists (H2RA) is recommended by the gastroenterological society guidelines and SSc specific guidelines [13, 14]. Whether these medications impact ILD development or progression is unknown, with studies examining the benefit of anti-reflux medications (such as PPI and H2RA) in idiopathic pulmonary fibrosis (IPF) producing conflicting results [15–19] even though these medications are conditionally recommended by ILD society guidelines [20]. While some studies show a survival benefit in IPF patients prescribed anti-reflux medication (PPI or H2RA), the majority of these studies have been influenced by immortal time bias [17]. Additionally, assessment of the effect of anti-reflux medication on IPF progression has produced mixed findings [15, 18]. There is scarce literature on the impact of anti-reflux medication in SSc-ILD, with a recent large German study (*n* = 1931 SSc patients) showing that PPI use in SSc-ILD significantly improved overall survival and progression free survival over 5 years [21]. No other reflux treatments such as H2RA, were evaluated in the German study.

The aim of our study was to evaluate the relationship between SSc-ILD and GORD in Australian SSc patients and assess the impact of anti-reflux medications (PPI and H2RA) on outcomes and survival in SSc-ILD.

## Methods

### Study population

Consecutive SSc patients from the Australian Scleroderma Cohort Study (ASCS) were included in this analysis (Table 1). The ASCS, established in 2007, is a prospective multi-centre cohort study of risk and prognostic factors for SSc. The ASCS consists of SSc patients who fulfill the 2013 ACR/EULAR criteria for SSc [22] and attend yearly screening for ILD and pulmonary arterial hypertension (PAH) with pulmonary function tests (PFTs) and transthoracic echocardiography (TTE). Data from this cohort is collected annually and includes patient demographics, SSc disease features and patient reported outcome measures. Each participant gives consent to be part of the ASCS with ethical approval obtained from all the participating study sites.

**Table 1:** Patient demographics and clinical characteristics

**Characteristics (*n* = number for whom data available)**	***n* = 1632** **(mean ± SD, n(%))**
Age at SSc onset, years **(*n* = 1549)**	47.4 (36.6-57.1)
Female **(*n* = 1629)**	1391 (85%)
Disease duration, years (*n* = 1549)	7.4 (2.7-15.7)
Disease subtype (n = 1572)	
Limited	1171 (74%)
Diffuse	409 (26%)
ANA centromere ( +) (*n* = 1579)	723 (46%)
Scl-70 ( +) (n = 1560)	232 (15%)
U1RNP ( +) (*n* = 1559	103 (7%)
RNA polymerase 3 ( +) (*n* = 1087)	156(14%)
Highest mRSS (*n* = 1608)	8 (5-16)
Joint Contractures (*n* = 1614)	684 (42%)
Digital Ulcers (*n* = 1631)	746 (45%)
Calcinosis (*n* = 1063)	81 (8%)
GIT manifestations	
GORD (*n* = 1632)	1539 (94%)
Dysphagia (*n* = 1373)	840 (61%)
Diarrhoea (*n* = 1621)	831 (51%)
Constipation (*n* = 1620)	828 (51%)
SIBO (*n* = 1632)	53 (3%)
Anal incontinence (*n* = 1622)	503 (31%)
Pseudo-obstruction (*n* = 1564)	61 (4%)
Myositis (*n* = 1632)	1525 (93%)
Myocardial disease (*n* = 1632)	115 (7%)
PAH (*n* = 1632)	216 (13%)
ILD (*n* = 717)	469 (29%)
ILD severity (*n* = 444)	
Mild (< 20%)	248 (56%)
Moderate (20-30%)	105 (24%)
Severe (> 30%)	91 (21%)

*Abbreviations*: *SSc* systemic sclerosis, *SD* standard deviation, *GIT* Gastrointestinal, *GORD* gastroesophageal reflux disease, *mRSS* modified Rodnan skin score, *PAH* pulmonary arterial hypertension, *ILD* interstitial lung disease, *HRCT* High Resolution Computed Tomography, *SIBO* small intestinal bacterial overgrowth, *PPI* proton pump inhibitor, *H2RA* histamine 2 receptor antagonist

PAH defined as ≥ 20 mmHg and a pulmonary capillary wedge pressure (PCWP) ≤ 15 mmHg and pulmonary vascular resistance (PVR) ≥ 3 Woods units on right heart catheter

ILD defined as the presence of characteristic pulmonary fibrosis on HRCT of the chest

ILD severity categorised as mild (fibrosis involving < 20% of the lung fields), moderate (fibrosis involving 20-30% of the lung fields) and severe (fibrosis involving > 30% of the lung fields)

### Data collection

The following prospectively collected data from the ASCS were included in this analysis: demographic data (age and sex) and disease-related data including disease duration (defined as the time since the onset of the first non-Raynaud’s disease manifestation), disease subtype, modified Rodnan skin score (mRSS), autoantibody status and disease treatment. SSc disease complications recorded included ILD, PAH, myocardial disease, myositis, Raynaud’s phenomenon, gastrointestinal symptoms, digital ulceration, and joint contractures. GORD was defined as the presence of at least one of the following criteria: patient-reported symptoms of reflux, endoscopic evidence of reflux oesophagitis and/or treatment with a PPI and/or H2RA. ILD was defined by the presence of characteristic pulmonary fibrosis on HRCT. High resolution computed tomography were undertaken in patients with clinical suspicion for ILD (including abnormalities on PFT or clinical findings consistent with ILD). ILD severity was defined by the extent of pulmonary fibrosis seen on HRCT chest and categorised as mild (fibrosis involving < 20% of the lung fields), moderate (fibrosis involving 20–30% of the lung fields) and severe (fibrosis involving > 30% of the lung fields) with the worst ever recorded severity included in this analysis. PAH was defined by right heart catheterisation according to international criteria [23]. Myositis was defined as present if all the following were recorded: (i) muscle weakness on examination;(ii) elevated creatine kinase above baseline and (iii) myopathic changes on electromyography (EMG) or evidence of muscle inflammation on magnetic resonance imaging (MRI) or muscle biopsy. Cardiac involvement was defined as present if systolic or diastolic dysfunction was noted on TTE or if a physician had attributed the presence of conduction abnormalities to SSc. Small intestinal bacterial overgrowth (SIBO) was defined as the presence of new diarrhoea improved by cyclical antibiotic therapy. Gastrointestinal symptoms recorded included dysphagia, diarrhoea, bloating, constipation and faecal incontinence.

### Outcomes

The association between patient demographic and disease features as well as SSc complications and the presence of GORD was evaluated. In SSc-ILD patients, the relationship between the presence of GORD and ILD severity (defined as % fibrosis measured on HRCT of the chest); time to development of ILD (from onset of first non-Raynaud’s SSc disease manifestation) and impact of GORD treatment (with a PPI or combination PPI/H2RA) on survival were evaluated.

### Statistical analysis

All statistical analyses were performed using STATA 15.1 (StataCorp LP, College Station, TX, USA). Data are presented as mean ± standard deviation (SD) for normally distributed variables and median (interquartile range (IQR)) for non-normally distributed continuous variables, and as number (percentage) for categorical variables. Differences in frequency were tested using chi-square and Fisher’s exact tests. For normally distributed continuous variables p-value was calculated using Students T-test. For non-normally distributed continuous variables p-value was calculated using Wilcoxon rank-sum test. A p-value of < 0.05 was considered statistically significant. Time to ILD development and survival were analysed using Kaplan-Meier (K-M) survival curves. Multivariable analyses were performed using Cox-regression analyses adjusting for disease duration, disease subtype, ILD treatment with mycophenolate or cyclophosphamide and co-existent PAH. Propensity score analysis was performed in SSc-ILD to estimate treatment effect and reduce bias. Models were created with variables consisting of clinically significant determinants of GORD (based on expert opinion) including diffuse disease subtype, forced vital capacity (FVC) < 70%, hospitalisation and dysphagia. No variable had greater than 5% bias. Cox-regression analyses were then performed on propensity matched cohorts to investigate the effect of combination versus single agent GORD treatment on survival adjusting for ILD treatment with mycophenolate or cyclophosphamide, and co-existent PAH.

## Results

### Demographic and disease features by GORD status

Data from 1632 consecutive SSc patients were included in this analysis, with patient characteristics summarised in Table 1. Most patients were female (85%) with limited disease subtype (74%). Anti-centromere autoantibody positivity was seen in 46% of patients with 15% positive for anti-Scl-70 antibody.

### Demographic and disease features by GORD status

GORD affected 1539 (94%) of the SSc cohort. When comparing those with GORD to those without, there was no significant difference in age, sex, disease subtype, or autoantibody profile (Supplementary Table 1). Those with GORD compared to those without GORD had a longer SSc disease duration (7.3 vs 3.8 years, *p* = 0.001), a higher mRSS (eight vs six, *p* = 0.0004), and a higher frequency of joint contractures (43% vs 25%, *p* = 0.0004) and digital ulceration (47% vs 26%, *p* < 0.0001). The was no difference in the frequency of cardiopulmonary manifestations (PAH, ILD, or myocardial involvement), myositis, or calcinosis. In terms of GI manifestations, those with GORD compared to those without GORD were more likely to report a range of other gastrointestinal manifestations including dysphagia, diarrhoea, constipation, and faecal incontinence. The presence of GORD was not associated with development of SIBO in our cohort.

Of patients with GORD, 1374 (90%) were on treatment with either a PPI or H2RA or combination therapy. Most patients with GORD used PPIs (90%) and 362 (23%) were prescribed H2RA. Three hundred and fifty-one (23%) participants were on combination therapy with both PPI and H2RA.

### GORD and SSc-ILD

GORD affected 450 (96%) of the SSc-ILD cohort. When comparing participants with ILD and GORD to those without, there was no significant difference in sex or autoantibody profile (Table 2). Those with GORD compared to those without GORD were younger (47.5 vs 58.3 years, *p* = 0.016), had a higher mRSS (ten vs four, *p* = 0.0003) and a higher frequency of diffuse disease subtype (41% vs 11%, *p* = 0.0071), joint contractures (55% vs 32%, *p* = 0.046), and digital ulceration (55% vs 32%, *p* < 0.05). Participants with GORD were less likely to have PAH (16% vs 37%, *p* = 0.014). There was no significant association between GORD and the presence of ILD (66% vs 59%, *p* = 0.46, Supplementary Table 1), or time to ILD development (*p* = 0.29, See Supplementary Fig. 1). Participants with GORD were not found to have more severe ILD on HRCT (see Table 2, mild 56% vs 50%, moderate 24 vs 28%, and severe 20% vs 22% *p* = 0.87).

**Table 2:** Demographic and clinical characteristics of the SSc-ILD cohort by GORD status

**Characteristics (*n* = number for whom data available)**	**GORD (*n* = 450)** **(mean ± SD, n(%))**	**No GORD (*n* = 19)** **(mean ± SD, n(%))**	**p**
Age at SSc onset, years (*n* = 453)	47.5 (35.7-56.4)	58.3 (41.4-65.7)	0.016
Female (*n* = 469)	361 (80%)	13 (68%)	0.21
Disease duration, years (*n* = 453)	6.7 (2.1-15.6)	5.0 (2.0-19.4)	0.95
Disease subtype (*n* = 451)			
Limited	253 (59%)	17 (89%)	
Diffuse	179 (41%)	2 (11%)	0.0071
ANA centromere ( +) (*n* = 460)	81 (18%)	2 (11%)	0.38
Scl-70 ( +) (*n* = 457)	147 (33%)	5 (28%)	0.61
U1RNP ( +) (*n* = 457)	35 (8%)	0 (0%)	0.38
RNA polymerase 3 ( +) (*n* = 355)	54 (16%)	1 (7%)	0.38
Highest mRSS (*n* = 468)	10.0 (6.0-21.0)	4.0 (2.0-9.0)	0.0003
Joint Contractures (*n* = 467)	246 (55%)	6 (32%)	0.046
Digital Ulcers (*n* = 467)	249 (55%)	6 (32%)	0.042
Calcinosis (*n* = 356)	22 (6%)	2 (12%)	0.40
GIT manifestations			
Dysphagia (*n* = 417)	258 (64%)	2 (14%)	0.0002
Diarrhoea (*n* = 468)	254(56%)	3 (17%)	0.0009
Constipation (*n* = 468)	238 (53%)	3 (17%)	0.0026
SIBO (*n* = 469)	17 (4%)	0 (0%)	0.39
Faecal incontinence (*n* = 469)	144 (32%)	0 (0%)	0.0031
Pseudo-obstruction (*n* = 457)	23 (5%)	0 (0%)	0.31
Myositis (*n* = 469)	55 (12%)	2 (11%)	0.82
Myocardial disease (*n* = 469)	58 (13%)	1 (5%)	0.33
PAH (*n* = 469)	70 (16%)	7 (37%)	0.014
ILD severity (*n* = 443)			
Mild (< 20%)	238 (56%)	9 (50%)	0.87
Moderate (20-30%)	100 (24%)	5 (28%)	
Severe (> 30%)	87 (20%)	4 (22%)	
GORD Treatment (*n* = 469)			
PPI use	418 (92%)	0 (0%)	< 0.0001
H2RA use	121 (27%)	0 (0%)	0.0087
Combination therapy	120 (27%)	0 (0%)	< 0.0001

*Abbreviations*: *SSc* systemic sclerosis, *SD* standard deviation, *GIT* gastrointestinal, *GORD* gastroesophageal reflux disease, *mRSS* modified Rodnan skin score, *PAH* pulmonary arterial hypertension, *ILD* interstitial lung disease, *HRCT* High Resolution Computed Tomography, *SIBO* small intestinal bacterial overgrowth, *PPI* proton pump inhibitor, *H2RA* histamine 2 receptor antagonist

PAH defined as ≥ 20 mmHg and a pulmonary capillary wedge pressure (PCWP) ≤ 15 mmHg and pulmonary vascular resistance (PVR) ≥ 3 Woods units on right heart catheter

ILD defined as the presence of characteristic pulmonary fibrosis on HRCT of the chest

ILD severity categorised as mild (fibrosis involving < 20% of the lung fields), moderate (fibrosis involving 20-30% of the lung fields) and severe (fibrosis involving > 30% of the lung fields)

### Effect of treatment of GORD on survival in SSc-ILD

**Fig. 1:**
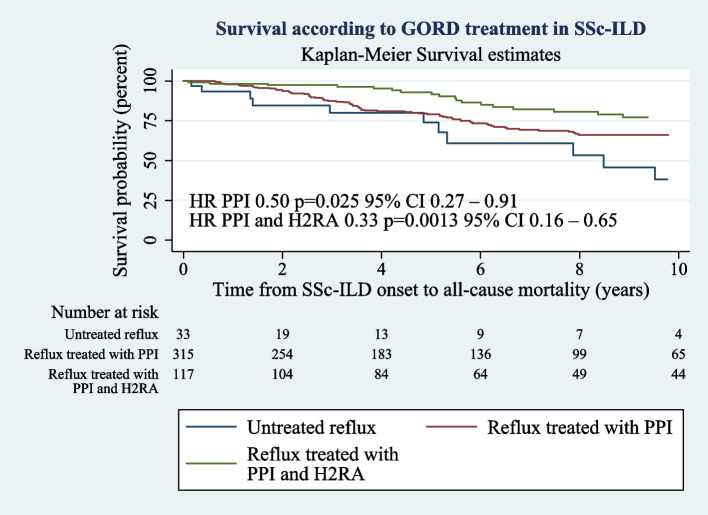
Shows the Kaplan-Meier survival curve from date of ILD onset to all-cause mortality by GORD treatment agent for patients with GORD

In participants with GORD and ILD, 66% were prescribed single agent therapy, 27% were on combination PPI and H2RA, while 7% were not prescribed anti-reflux agents. Treatment of GORD with PPI alone was associated with significantly improved survival in participants with SSc-ILD (HR 0.5, *p* = 0.025, CI 0.3-0.9, see Supplementary Table 2) on univariate analysis and on multivariable analysis (HR 0.5, CI 0.3 to 0.9, *p* = 0.031, Table 3, Fig. 1). In participants with ILD, more aggressive GORD treatment with both H2RA and PPI was associated with improved survival over single agent therapy (HR 0.3 CI 0.2-0.7, *p* = 0.0013) on univariate analysis. This effect remained significant in multivariable analysis after adjusting for disease subtype, disease duration, presence of co-existent PAH, ILD severity, and treatment of ILD (HR 0.3, CI 0.2-0.6, *p* = 0.0009 see Table 3). Multivariable cox regression analysis using the propensity adjusted cohort also showed improved survival in participants on combination therapy compared to single agent treatment (HR 0.3, CI 0.2 to 0.5, *p* < 0.0001, see Supplementary Table 3).

**Table 3:** Multivariable model from SSc-ILD diagnosis to all-cause mortality

**Variable**	**Hazard ratio**	**p**	**95% CI**
**GORD treatment status**			
No treatment	1.0		
Single agent treatment (PPI)	0.5	0.031	0.3-0.9
Combination treatment (PPI + H2RA)	0.3	0.0009	0.2-0.6
**PAH**	4.3	< 0.0001	2.9-6.2
**ILD treatment status**			
Mycophenolate^a^	0.5	0.0093	0.3-0.9
Cyclophosphamide^b^	1.3	0.15	0.9-2.0

*GORD* gastroesophageal reflux disease, *PPI* Proton Pump Inhibitor, *H2RA* Histamine 2 Receptor Antagonist, *PAH* Pulmonary Arterial Hypertension by international definition mean PAP >  = 20 AND PCWP <  = 15 AND PVR Woods unit > 3 Woods units on right heart catheter, *ILD* interstitial lung disease defined as the presence of characteristic pulmonary fibrosis on HRCT of the chest

^a^No treatment with mycophenolate used as reference

^b^No treatment with cyclophosphamide used as reference

## Discussion

Our study shows that the use of anti-reflux medication for the management of GORD in SSc-ILD is associated with a survival benefit. Our findings are supported by a recent study using data from 1931 participants with SSc-ILD from the German Network for Systemic Sclerosis (DNSS). In this study, the use of PPIs was associated with an improved 5 year survival and 5 year progression free survival in participants with SSc-ILD (91% vs 71%, *p* < 0.0001 and 67% vs 46%, *p* < 0.0001 respectively) on both univariate and multivariable analysis [21]. Our study found that single agent therapy with PPI was associated with improved survival in SSc-ILD on univariate analysis (HR 0.5, *p* = 0.025) and multivariable analysis (HR 0.5, *p* = 0.031) adjusted for covariates such as disease duration, disease subtype, ILD treatment with mycophenolate or cyclophosphamide, and co-existent PAH. Additionally, we found that aggressive management of GORD in SSc-ILD (with combination PPI and H2RA) was associated with improved survival compared to single agent therapy with PPI alone. Studies have shown that maximal dose PPI is unable to fully control GORD in 50-60% of SSc patients [24, 25]. There are also a number of studies which show that addition of H2RA to high dose PPI in the general population improves suppression of intragastric acid (defined as pH > 4) particularly overnight [26, 27]. It is therefore plausible that the addition of H2RA to PPI therapy can improve GORD control in SSc patients, (particularly overnight during prolonged periods in the supine position), thereby reducing micro aspiration and resulting in the improved survival seen in those prescribed dual therapy.

In our cohort, GORD was not associated with an increased risk of ILD development or severity. When looking at the association between GORD and SSc-ILD, the literature is conflicting, possibly due to the differences in how GORD is defined. While some studies use imaging modalities or motility studies to diagnose GORD, other studies rely on patient reported symptoms. Several studies where GORD is defined through motility studies or the presence of oesophageal dilatation on HRCT scan, demonstrate an association between GORD and SSc-ILD [5, 10–12] A recently published post-hoc analysis of the Scleroderma Lung Study (SLS) II examined the association between patient reported reflux symptoms and ILD severity defined by FVC or radiographic measures [28]. This study found that the severity of reported reflux symptoms (as measured by the UCLA GIT 2.0 questionnaire) was not associated with ILD severity at baseline but did significantly correlate with radiographic progression over 2 years. Additionally, the results presented by Kreuter et al. [21], using data from 1931 participants with SSc-ILD, found that the presence of GORD (defined by patient reported symptoms) was not associated with worse 5 year survival (79% vs 83%, *p* = 0.36) or worse progression free survival (62% vs 68%, *p* = 0.57), as was seen in our analysis. Our definition of GORD was based primarily on patient reported symptoms, endoscopic findings and the use of GORD therapy. As studies show that many SSc patients have evidence of dysmotility or acid reflux on manometry in the absence of symptoms [12], it is possible these patients were not included in our analysis. As we used the worst-ever reported ILD scores for our analysis, we are unable to comment on the relationship between reflux severity and ILD progression.

The treatment of GORD (with PPI or combination of PPI and H2RA) was not associated with delayed ILD development in our SSc cohort. This finding is consistent with a recently published study of 798 Canadian SSc patients followed over 4.4 years [29], where patients exposed to gastroprotective agents did not have a lower incidence of ILD over the follow up period compared to the unexposed population. Asymptomatic oesophageal dysmotility is often present early in the SSc disease course and occasionally prior to SSc diagnosis [11, 12]. Therefore, the lack of observed effect of reflux treatment on development of SSc-ILD may be due to the accumulated damage from aspiration prior to GORD diagnosis and initiation of its treatment.

The evidence for use of anti-reflux therapy to improve survival or delay ILD disease progression in idiopathic pulmonary fibrosis (IPF) is mixed [15–19]. One recent study of patients in an Australian IPF registry found that neither GORD severity nor use of PPI/ H2RA improved survival or delayed disease progression [15]. This study boasted large patient numbers (*n* = 684) and long follow up (median 2.2 years). However, while 65% of patients were taking PPI/H2RA, only 41% had a diagnosis of GORD, which is in contrast to our study.

Increasing awareness of the potential adverse effects of PPIs has led to some uncertainty about their use in SSc [8]. A recent review article discussed the difficulties in balancing the benefit of PPI use in SSc with potential adverse effects. The most common side effects reported with PPI use are osteoporosis, hypomagnesemia and atrophic gastritis [8]. While the use of PPIs has been associated with development of SIBO in SSc, due to decreased acidity of gastric contents, we did not find PPI use to be associated with development of SIBO in our cohort [30]. This may be due to the low numbers of participants reported to have SIBO in our cohort (3.2%). Additionally, due to the low numbers of participants not treated with PPI (17% overall), we cannot ascertain the association between PPI use and the adverse effects listed above in our cohort. Whilst we are not recommending the empiric use of anti-reflux therapy in all SSc patients, we are highlighting that in those with GORD and SSc-ILD, the treatment of GORD with PPI or combination PPI/H2RA is associated with improved survival, and in this context, benefits of treatment may outweigh potential risks. We acknowledge that the H2RA ranitidine has been withdrawn from the market due to the presence of N-nitrosodimethylamine (NDMA). However, other H2RA such as nizatidine remain available.

Strengths of our study include the large well characterised SSc cohort, with data from 1640 participants undergoing yearly screening for SSc-ILD. Limitation of our study include immortal time bias, as patients who live longer are more likely to have been prescribed anti-reflux medication; some missing data; and the inability to ascertain the dosage of anti-reflux medication used or participants’ compliance with treatment. As the practice of Australian physicians is generally to optimise PPI therapy in SSc prior to initiation of H2RA (in the absence of contra-indications), participants on combination therapy may have been prescribed higher doses of PPI than participants on PPI alone. Additionally, we do not routinely perform manometry or endoscopy in Australian SSc patients; therefore, participants with asymptomatic dysmotility or reflux would not have been included in our GORD population. However, some patient may have been diagnosed incidentally on gastroscopy performed for other indications.

## Conclusion

GORD is a common complication in SSc, affecting 94% of Australian SSc patients. Treatment of GORD with either PPI or combination PPI/H2RA has the potential to improve survival in those with SSc-ILD.

### Supplementary Information

Supplementary Material 1.

## Data Availability

The datasets used and/or analysed during the current study are available from the corresponding author on reasonable request.

## Abbreviations

ACR: American College of Rheumatology
ASCS: Australian Scleroderma Cohort Study
CI: Confidence interval
EMG: Electromyography
EULAR: European League Against Rheumatism or European Alliance of Associations for Rheumatology
FVC: Forced vital capacity
GI: Gastrointestinal
GIT: Gastrointestinal tract
GORD: Gastroesophageal reflux disease
H2RA: Histamine receptor antagonist
HR: Hazard ratio
HRCT: High resolution computed tomography
ILD: Interstitial lung disease
IPF: Idiopathic pulmonary fibrosis
IQR: Interquartile range
K-M: Kaplan:Meier
LES: Lower oesophageal sphincter
MRI: Magnetic resonance imaging
mRSS: Modified Rodnan skin score
PAH: Pulmonary Arterial Hypertension
PFTs: Pulmonary function tests
PPI: Proton pump inhibitor
SIBO: Small intestinal bacterial overgrowth
SSc: Systemic Sclerosis
TTE: Transthoracic echocardiography
UCLA: University of California and Los Angeles
