# Supplementary material for: The impact of gastroesophageal reflux disease and its treatment on interstitial lung disease outcomes
Source: Arthritis Res Ther. 2024 Jun 25;26:124. doi: 10.1186/s13075-024-03355-0 (PMC11197189; doi:10.1186/s13075-024-03355-0)
Supplement: Supplementary file 1 — Supplementary Material 1. [file 13075_2024_3355_MOESM1_ESM.docx]

Supplementary Table of Contents

Supplementary table 1: Patient demographic and clinical characteristics by GORD status page 2

Supplementary table 2: Univariable model for survival from SSc-ILD diagnosis to

all-cause mortality page 3

Supplementary figure 1: Kaplan-Meier survival estimates from SSc onset to ILD onset by

GORD treatment status page 4

Supplementary table 3: SSc-ILD patient demographic and autoantibody characteristics by GORD page 5

treatment status

Supplementary figure 2: Histogram of propensity scores in SSc-ILD patients treated with single page 6 agent versus combination reflux treatment

Supplementary table 4: Multivariable model for survival from SSc-ILD diagnosis to all-cause page 6 mortality in the propensity matched cohort

**Supplementary table 1: Patient demographic and clinical characteristics by GORD status**

| **Characteristics** (n=number for whom data available) | **GORD (n=1531)**  **(mean ± SD, n(%))** | **No GORD (n=101)**  **(mean ± SD, n(%))** | **p value** |
| --- | --- | --- | --- |
| Age at SSc onset, years (n=1549) | 47.4 (36.6-56.9) | 48.9 (38.9-59.5) | 0.13 |
| Female (n=1629) | 1300 (85%) | 84 (83%) | 0.60 |
| Disease duration, years (n=1549) | 7.3 (2.7-15.7) | 3.8 (1.7-11.8) | 0.0011 |
| Disease subtype (n=1572)  Limited  Diffuse | 1086 (74%)  387 (26%) | 81 (82%)  18 (18%) | 0.075 |
| ANA centromere (+) (n =1579) | 684 (46%) | 37 (39%) | 0.15 |
| Scl-70 (+) (n=1560) | 215 (15%) | 16 (17%) | 0.47 |
| U1RNP (+) (n=1559) | 98 (7%) | 5 (5%) | 0.64 |
| RNA polymerase 3 (+) (n=1087) | 149 (15%) | 7 (11%) | 0.37 |
| Highest mRSS (n=1608) | 8.0 (5.0-16.0) | 6.0 (3.0-12.0) | 0.0004 |
| Joint Contractures (n=1614) | 657 (43%) | 25 (25%) | 0.0004 |
| Digital Ulcers (n=1631) | 718 (47%) | 26 (26%) | <0.0001 |
| Calcinosis (n=1063) | 79 (8%) | 2 (3%) | 0.17 |
| GIT manifestations  Dysphagia (n=1373)  Diarrhoea (n=1621)  Constipation (n=1620)  SIBO (n=1632)  Faecal incontinence (n=1622)  Pseudo-obstruction (n=1564) | 830 (64%)  801 (53%)  801 (53%)  52 (3%)  491 (32%)  61 (4%) | 8 (11%)  26 (26%)  24 (24%)  1 (1%)  11 (11%)  0 (0%) | <0.0001  <0.0001  <0.0001 0.19  <0.0001  0.043 |
| Myositis (n=1632) | 110 (7%) | 5 (5%) | 0.40 |
| Myocardial disease (n=1632) | 140 (9%) | 5 (5%) | 0.15 |
| PAH (n=1632) | 163 (11%) | 11 (11%) | 0.94 |
| ILD severity (n=423)  Mild (<20%)  Moderate (20-30%)  Severe (>30%) | 240 (56%)  101 (24%)  101 (24%) | 7 (47%)  4 (27%)  4 (27%) | 0.75 |
| GORD treatment (n=1632)  PPI use  H2RA use  Combination therapy | 1374 (90%)  354 (23%)  351 (23%) | 0 (0.0%)  0 (0.0%)  0 (0.0%) | <0.0001  <0.0001  <0.0001 |

*Abbreviations: SSc- systemic sclerosis, SD- standard deviation, GIT- gastrointestinal, GORD- gastroesophageal reflux disease, mRSS- modified Rodnan skin score, PAH- pulmonary arterial hypertension, ILD – interstitial lung disease, HRCT- High Resolution Computed Tomography, SIBO- small intestinal bacterial overgrowth, PPI- proton pump inhibitor, H2RA- histamine 2 receptor antagonist*

*PAH defined as >20mmHg and a pulmonary capillary wedge pressure (PCWP) <15mmHg and pulmonary vascular resistance (PVR) > 3 Woods units on right heart catheter*

*ILD defined as the presence of characteristic pulmonary fibrosis on HRCT of the chest*

*ILD severity categorised as mild (fibrosis involving <20% of the lung fields), moderate (fibrosis involving 20- 30% of the lung fields) and severe (fibrosis involving >30% of the lung fields)*

**Supplementary table 2: Univariable model for survival from SSc-ILD diagnosis to all-cause mortality**

| Variable | Hazard ratio | p value | 95% CI |
| --- | --- | --- | --- |
| **GORD treatment status*** |  |  |  |
| **Single agent treatment**  **(PPI)** | 0.5 | 0.025 | 0.3-0.9 |
| **Combination treatment (PPI + H2RA)** | 0.3 | 0.0013 | 0.2-0.7 |

*GORD- gastroesophageal reflux disease, PPI- Proton Pump Inhibitor, H2RA- Histamine 2 Receptor Antagonist*

**No treatment used as reference*

**Supplementary figure 1: Kaplan-Meier survival estimates from SSc onset to ILD onset by GORD treatment status**

**
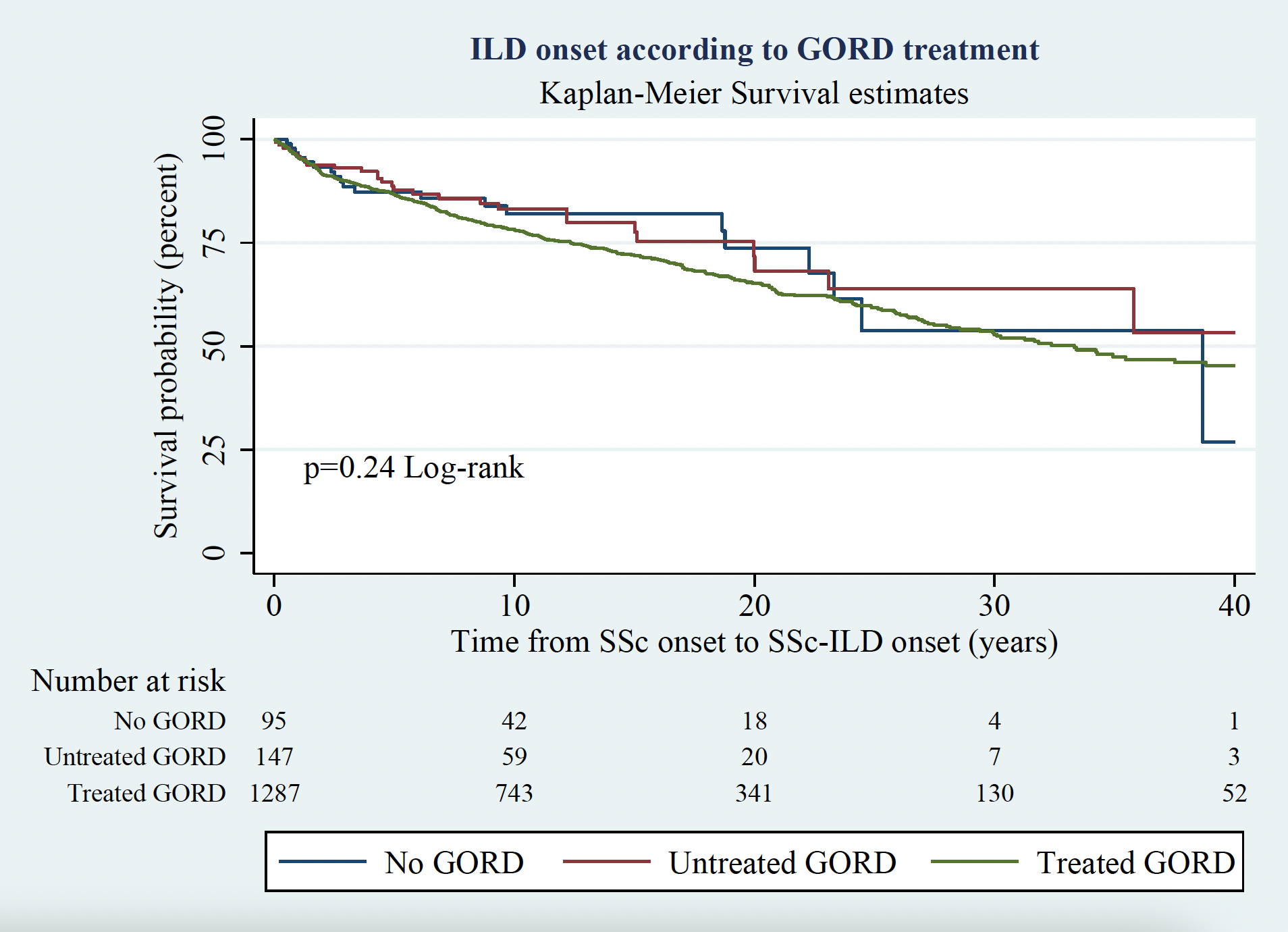
**

**Supplementary table 3: SSc-ILD patient demographic and autoantibody characteristics by GORD treatment status**

| **Characteristics** (n=number for whom data available) | **Combination treatment (H2RA+ PPI) (n=117)** | **Single agent treatment (PPI) (n=297)** | **No treatment**  **(n=32)** | **p-value** |
| --- | --- | --- | --- | --- |
| Age at SSc onset, years (n=433) | 46.5 (34.4-54.7) | 47.6 (36.4-57.3) | 46.1 (36.0-56.2) | 0.33 |
| Female (n=448) | 98 (82%) | 237 (80%) | 25 (78%) | 0.79 |
| Death (n=448) | 28 (24%) | 77 (26%) | 11 (34%) | 0.46 |
| Disease subtype (n=430)  Limited  Diffuse | 68 (59%)  47 (41%) | 167 (59%)  118 (41%) | 17 (57%)  13 (43%) | 0.97 |
| ANA centromere (+) (n=439) | 24 (20%) | 56 (19%) | 1 (3%) | 0.066 |
| Scl-70 (+) (n=437) | 42 (37%) | 93 (32%) | 12 (38%) | 0.62 |
| U1RNP (+) (n=437) | 8 (7%) | 25 (9%) | 2 (6%) | 0.80 |
| RNA polymerase 3 (+) (n=340) | 13 (15%) | 37 (16%) | 4 (16%) | 0.93 |
| Highest mRSS (n=447) | 12.0 (7.0-24.0) | 10.0 (6.0-21.0) | 10.5 (6.0-22.0) | 0.24 |
| Joint Contractures (n=446) | 75 (63%) | 154 (52%) | 15 (47%) | 0.088 |
| Digital Ulcers (n=448) | 75 (63%) | 157 (53%) | 15 (47%) | 0.11 |
| Calcinosis (n=338) | 9 (10%) | 11 (5%) | 2 (9%) | 0.24 |
| GIT manifestations  Dysphagia (n=401)  Diarrhoea (n=448)  Constipation (n=448)  SIBO (n=448)  Faecal incontinence (n=448)  Pseudo-obstruction (n=436) | 83 (73%)  75 (63%)  78 (66%)  9 (8%)  54 (45%)  10 (9%) | 167 (64%)  168 (57%)  148 (50%)  8 (3%)  86 (29%)  11 (4%) | 6 (25%)  9 (28%)  10 (31%)  0 (0%)  3 (9%)  1 (3%) | <0.001  0.002  0.001  0.032  <0.001  0.13 |
| Myositis (n=448) | 16 (13%) | 36 (12%) | 2 (6%) | 0.54 |
| Myocardial disease (n=1632) | 18 (15%) | 38 (12%) | 2 (6%) | 0.41 |
| PAH (n=423) | 24 (20%) | 57 (19%) | 5 (16%) | 0.85 |
| ILD severity (n=423)  Mild (<20%)  Moderate (20-30%)  Severe (>30%) | 66 (58%)  22 (20%)  25 (22%) | 155 (55%)  68 (24%)  59 (21$) | 17 (61%)  9 (32%)  9 (7%) | 0.33 |

*Abbreviations: SSc- systemic sclerosis, SD- standard deviation, GIT- gastrointestinal, GORD- gastroesophageal reflux disease, mRSS- modified Rodnan skin score, PAH- pulmonary arterial hypertension, ILD – interstitial lung disease, HRCT- High Resolution Computed Tomography, SIBO- small intestinal bacterial overgrowth, PPI- proton pump inhibitor, H2RA- histamine 2 receptor antagonist*

*PAH defined as >20mmHg and a pulmonary capillary wedge pressure (PCWP) <15mmHg and pulmonary vascular resistance (PVR) > 3 Woods units on right heart catheter*

*ILD defined as the presence of characteristic pulmonary fibrosis on HRCT of the chest*

*ILD severity categorised as mild (fibrosis involving <20% of the lung fields), moderate (fibrosis involving 20- 30% of the lung fields) and severe (fibrosis involving >30% of the lung fields)*

For categorical variables, a number (percentage) is presented with a p-value calculated by chi square test. For normally distributed continuous variables the mean (standard deviation) is presented with a p-value calculated by ANOVA. For non-normally distributed continuous variables the median (interquartile range) is presented with a p-value calculated by Kruskal-Wallis equality-of-populations rank test.

**Supplementary Figure 2: Histogram of propensity scores in SSc-ILD patients treated with single agent versus combination reflux treatment***
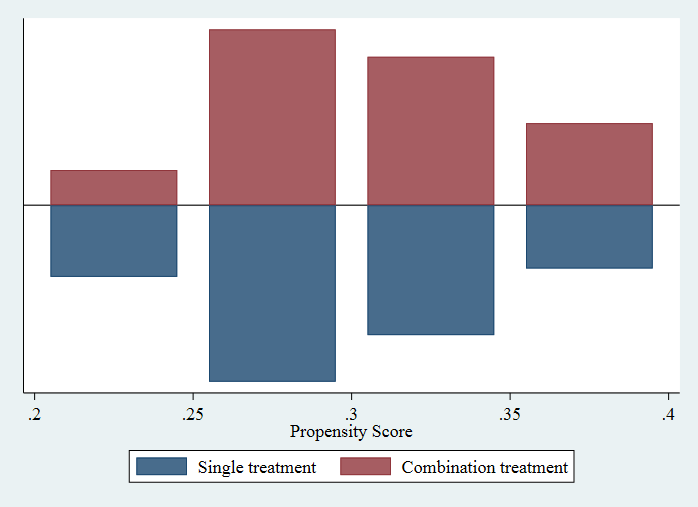


** Supplementary figure 2 is a histogram of propensity scores of the combination and single treatment groups, the distributions should match if the model is balanced. Models were created with variables consisting of clinically significant determinants of GORD (based on expert opinion) including diffuse disease subtype, forced vital capacity (FVC) <70%, hospitalization and dysphagia. No variable had greater than 5% bias.*

**Supplementary table 4: Multivariable model for survival from SSc-ILD diagnosis to all-cause mortality in the propensity matched cohort**

| **Variable** | **Hazard ratio** | **p** | **95% CI** |
| --- | --- | --- | --- |
| **GORD treatment status** | | | |
| Single agent treatment  (PPI) | 1.0 |  |  |
| Combination treatment (PPI + H2RA) | 0.3 | <0.0001 | 0.2-0.5 |
| **PAH** | 8.6 | <0.0001 | 4.9-15.2 |
| **ILD treatment status** | | | |
| Mycophenolate* | 0.9 | 0.63 | 0.5-1.6 |
| Cyclophosphamide** | 1.0 | 0.96 | 0.55-1.9 |

*GORD- gastroesophageal reflux disease, PPI- Proton Pump Inhibitor, H2RA- Histamine 2 Receptor Antagonist, PAH- Pulmonary Arterial Hypertension by international definition mean PAP >=20 AND PCWP <=15 AND PVR Woods unit >3 Woods units on right heart catheter, ILD- interstitial lung disease defined as the presence of characteristic pulmonary fibrosis on HRCT of the chest*

*No treatment with mycophenolate used as reference

**No treatment with cyclophosphamide used as reference
